# Corrosion type identification in flanged joints using recurrent neural networks on electrochemical noise measurements

**DOI:** 10.1038/s41529-025-00638-y

**Published:** 2025-07-15

**Authors:** Soroosh Hakimian, Abdel-Hakim Bouzid, Lucas A. Hof

**Affiliations:** Mechanical Engineering Department, École de technologie supérieure, Montreal, QC Canada

**Keywords:** Engineering, Mechanical engineering, Materials science

## Abstract

Bolted flanged joints are essential for connecting piping and process equipment but are vulnerable to localized corrosion that leads to sudden, unpredictable leaks. Electrochemical noise (EN) measurements can detect such corrosion, yet processing EN data is time-consuming and requires expertise. This study applies recurrent neural networks (RNNs) to automate corrosion type identification on flange surfaces using raw EN signals from spontaneous electrochemical reactions. In this work, supervised, hybrid, and unsupervised ML approaches are evaluated using experimentally obtained EN data. Among supervised models, the long short-term memory (LSTM) model achieves 93.62% accuracy. A hybrid method combining LSTM autoencoder features with a random forest classifier improves accuracy to 97.85%. An unsupervised method using LSTM autoencoder, principal component analysis, and k-means clustering also shows strong potential for real-time corrosion monitoring. Automated identification of corrosion types on flanged joints supports more effective material protection strategies, reducing the risk of failure in critical infrastructure.

## Introduction

Bolted flanged joints are extensively used to connect pipelines, pressure vessels, and different structural components in seawater desalination equipment, hydrocarbon processing, nuclear industries, and wind turbine industries. This type of connection allows disassembly of pipelines for maintenance or cleaning, but poses a risk of leakage failure especially when exposed to aggressive media and environments while operating at high pressures and temperatures^1^. Flange face corrosion is one of the most repeatable cause of leakage failure according to the literature^2^. Corrosion on flange faces arises when fluids penetrate gaps and leak paths formed at the gasket and flange interface. These gaps result from material degradation due to corrosion and aging and are further widened by joint loosening due to creep-relaxation effects^1,3,4^, rotation of the flange^5,6^, and flange face irregularities^7^. Localized corrosion, such as pitting and crevice corrosion, at the interface of the flange and gasket is a major cause of leakage failure in flanged gasketed joints^2,8–11^. Crevice corrosion is not easily detectable or visible at the flange-gasket interface, and due to its localized nature, it exhibits a higher corrosion rate compared to general corrosion by several orders of magnitude^12^. Corrosion of the flange surface becomes detectable only when a leak already occurs, necessitating pipeline shutdowns and resulting in the loss of revenue and costly resources. Therefore, detection and monitoring of the early stages of localized corrosion are critical to prevent extensive damage on such systems.

Electrochemical noise measurement (ENM) is a method that gains increasing attention in the field of electrochemical monitoring methods^13,14^. The spontaneous fluctuations in potential and current are stemming from the corrosion processes on the metal surface that can be measured by ENM^15^. This method is suitable for in situ corrosion monitoring without applying an external potential, and it can also be used to detect the type of corrosion^16^. ENMs are typically conducted using a zero-resistance ammeter (ZRA) mode, where the electrochemical potential noise (EPN) is recorded between a working and reference electrode. The electrochemical current noise (ECN) is measured as the galvanic current between two nominally identical working electrodes. Care is taken to minimize aliasing and instrument noise through appropriate filtering and sampling strategies^17^. Characterization of localized corrosion through current and potential signal monitoring is the most interesting application of ENM^18^. EN was shown to be effective in identifying localized corrosion mechanisms, as transient features in the signal can reflect the amplitude and frequency of corrosion events associated with specific forms such as pitting or crevice corrosion^19^. This technique is also a valuable tool in assessing the performance of protective coatings and corrosion inhibitors^20,21^. Indeed, ENM shows great potential as a non-destructive monitoring tool; however, distinguishing between localized and general corrosion remains challenging because EN data is dependent on factors such as the electrode system type, electrode surface area, and the measurement technique used^22^.

In the literature, data analysis methods are typically categorized according to their operational domain, including time^23^, frequency, and time-frequency domain^24^. Obtaining appropriate feature variables and analytical approaches from the measured EN data to distinguish between different forms of corrosion during the monitoring is the main difficulty of this method^25^. Xia et al.^26^ demonstrated the use of EN for atmospheric corrosion monitoring by applying discrete wavelet transform (DWT) to extract time–frequency features related to corrosion forms. Their approach requires complex signal preprocessing such as DC component removal and careful interpretation of wavelet energy levels. The paper highlights the challenges associated with traditional EN signal analysis methods. In another study by Xia et al.^27^, combined EN analysis with Thevenin equivalent circuit modeling and fast Fourier transform (FFT) to investigate localized corrosion under dynamic seawater/air interface conditions. However, accurate interpretation can be challenging due to overlapping transient events and signal fluctuations, particularly when using large electrode surfaces. EN analysis has also been applied to monitor stress corrosion cracking (SCC) using advanced techniques such as wavelet energy distribution and chaos theory. The use of signal interpretation involves complex steps like DC removal, phase space reconstruction, and calculation of correlation dimension to characterize crack initiation and propagation stages^28^.

Recently, machine learning (ML) techniques, including deep learning (DL) approaches, have been increasingly utilized in the field of corrosion to analyze EN data for prediction or classification. Homborg et al.^29^ investigated the application of convolutional neural networks (CNN) for DL-based classification of images of the electrochemical noise time-frequency transient information from two types of pitting corrosion data. In this approach, two methods including continuous wavelet transform (CWT) spectra and modulus maxima (MM) are used to train the CNN. Their results show that training the CNN with the CWT and MM combination has a higher classification accuracy compared to using each method separately. In another study, Hou et al.^30^ extracted twelve features from the EN signals using a recurrent quantification analysis and they then classified the corrosion behavior to general, passive, and pitting corrosion using random forests (RF) and linear discriminant analysis (LDA). Nazarnezhad et al.^31^ used EN analysis parameters obtained from time domain, frequency domain, and time-frequency domain analysis methods as inputs in an artificial neural network (ANN) model and using galvanostatic electrochemical impedance spectroscopy as target values to determine the pitting stage in stainless steel 321. Furthermore, Alves et al.^32^ extracted features from EN data using wavelet transform and recurrence quantification analysis to train several ML techniques including the ANN type multilayer perceptron (MLP), probabilistic neural network (PNN), support vector machine (SVM), k-nearest neighbor (kNN), and decision tree (DT). Abdulmutaali et al.^33^ developed an unsupervised framework to monitor corrosion using EN measurements. They converted EN time-series signals into wavelet spectrogram images, extracted features using DL models (e.g., CNNs), and applied principal component analysis (PCA) for multivariate statistical process monitoring. Their method identified deviations from uniform corrosion without requiring labeled data, relying on image-based feature representations. Finally, Jian et al.^34^ deployed a feature vector of 10 elements obtained from the EN datasets as an input for training ANN and SVM models to distinguish the type of corrosion. Table 1 summarizes all ML and DL techniques that are used to analyze EN data for corrosion type classification.

**Table 1:** Summary of the ML and DL techniques used in analyzing EN data for corrosion type classification

Input features	Types of corrosion	ML or DL methods	Number of features	Reference
Images of the CWT spectrum and MM including transient locations	Pitting	CNN	Image size: 201 × 99 pixels, no manual feature vector	^29^
Recurrence quantification variables	General Pitting Passivation	LDA RF	12	^30^
Recurrence quantification variables	General Pitting Passivation	MLP	4	^43^
Time domain, frequency domain, time-frequency domain parameters	Pitting	ANN	26	^31^
R_n_, q, f_n_, energy of 7-level wavelet crystal	General Pitting Passivation	ANN SVM	10	^34^
Wavelet transform and recurrence quantification parameters	Crevice Passivation Pitting Watermark	MLP PNN kNN DT SVM	35	^32^
Wavelet spectrogram images of EN signals	General Pitting Passivation	LBP CNN PCA	59 (LBP) 2048 (CNN)	^33^

It can be concluded from the reviewed literature that ML and DL approaches used so far are promising, but require substantial amounts of labeled data to achieve accurate classification. This presents a major barrier for practical use in industrial applications, because collecting extensive labeled datasets in real-world corrosion environments is challenging. Additionally, these techniques are often limited by their dependence on feature vectors based on static signal characteristics, like noise resistance or frequency content, which may not adapt well to dynamic conditions in corrosion processes. While recent work by Abdulmutaali et al.^33^ has demonstrated the potential of unsupervised learning using image-based representations of EN signals, their approach still depends on transforming time-series signals into images and applying predefined segmentation, highlighting the need for alternative sequence-based unsupervised approaches that eliminate the need for predefined features or signal-to-image conversion.

Therefore, the main objective of this study is to investigate the potential of utilizing recurrent neural networks (RNN) for classifying EN data and to compare its accuracy with traditional ML techniques such as RF. RNNs are well-suited for time-series or sequential data as they can detect hidden patterns or recurring trends in nonlinear and dynamic datasets^35^. One of the key strengths of RNNs is their ability to retain information from previous hidden states, enabling the prediction of future outcomes^36^. This characteristic has made them widely adopted in fields like natural language processing and speech recognition^37,38^. Due to their recurrent structure, RNNs have the potential to be more flexible in handling variability within EN data compared to static classifiers.

This paper introduces three novel approaches using RNN models to classify corrosion types based on two input features; current and potential signals from EN data. In these developed approaches, firstly, labeled data obtained through controlled laboratory experiments, are used to train RNN models. Then, using these labeled data, a hybrid approach is used to improve the model’s performance. Finally, an unsupervised approach is proposed that is trained using unlabeled data, as mostly occurs in real-time corrosion monitoring.

To evaluate the classification performance of these models, different techniques including confusion matrix and other classification metrics, e.g., F1-score, precision, and recall are calculated. Indeed, the effectiveness of the different RNN-based methods for EN data analysis are validated by experimental corrosion data using an in-house developed bolted joint test rig^39^, highlighting their potential for real-time corrosion monitoring.

## Results and discussion

### Surface morphology and the corresponding noise signals

**Fig. 1:**
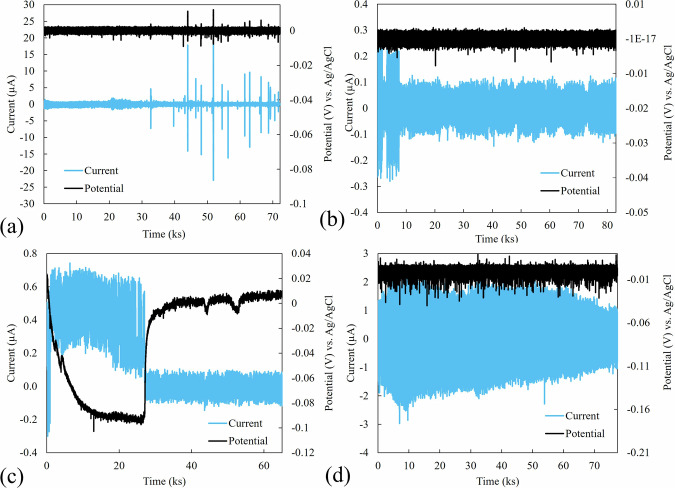
Measured EN signals. Electrochemical current and potential noise signals corresponding to the different types of corrosion occurred on the flange surface. **a** Pitting corrosion; (**b**) passive state; (**c**) crevice corrosion; (**d**) general corrosion.

The current (blue lines) and potential (black lines) noise signals obtained from the EN tests are presented in Fig. 1a–d. Figure 1a displays the transient signals associated with pitting corrosion on the flange sample plate. In pitting corrosion, the distinct current transients signify the initiation and progression of localized pits^40^. In the passive state (Fig. 1b), potential and current fluctuate steadily between −0.1 and 0.1 μA, except for the initial 10 ks, where fluctuations range from −0.3 to 0.3 μA. For crevice corrosion (Fig. 1c), noticeable transients in both current and potential signals indicate the initiation and propagation of crevice corrosion^41^. These transients are typically observed as rapid increases or decreases in the signals, depending on which W.E. is undergoing corrosion. In the case of general corrosion (Fig. 1d), the current fluctuations range between −3 and 2 μA, exceeding those of the passive state. The current and potential signals for pitting corrosion, general corrosion, and the passive state are detrended; however, the signals for crevice corrosion are not detrended to preserve the detection of transient events in the current and potential signals.

**Fig. 2:**
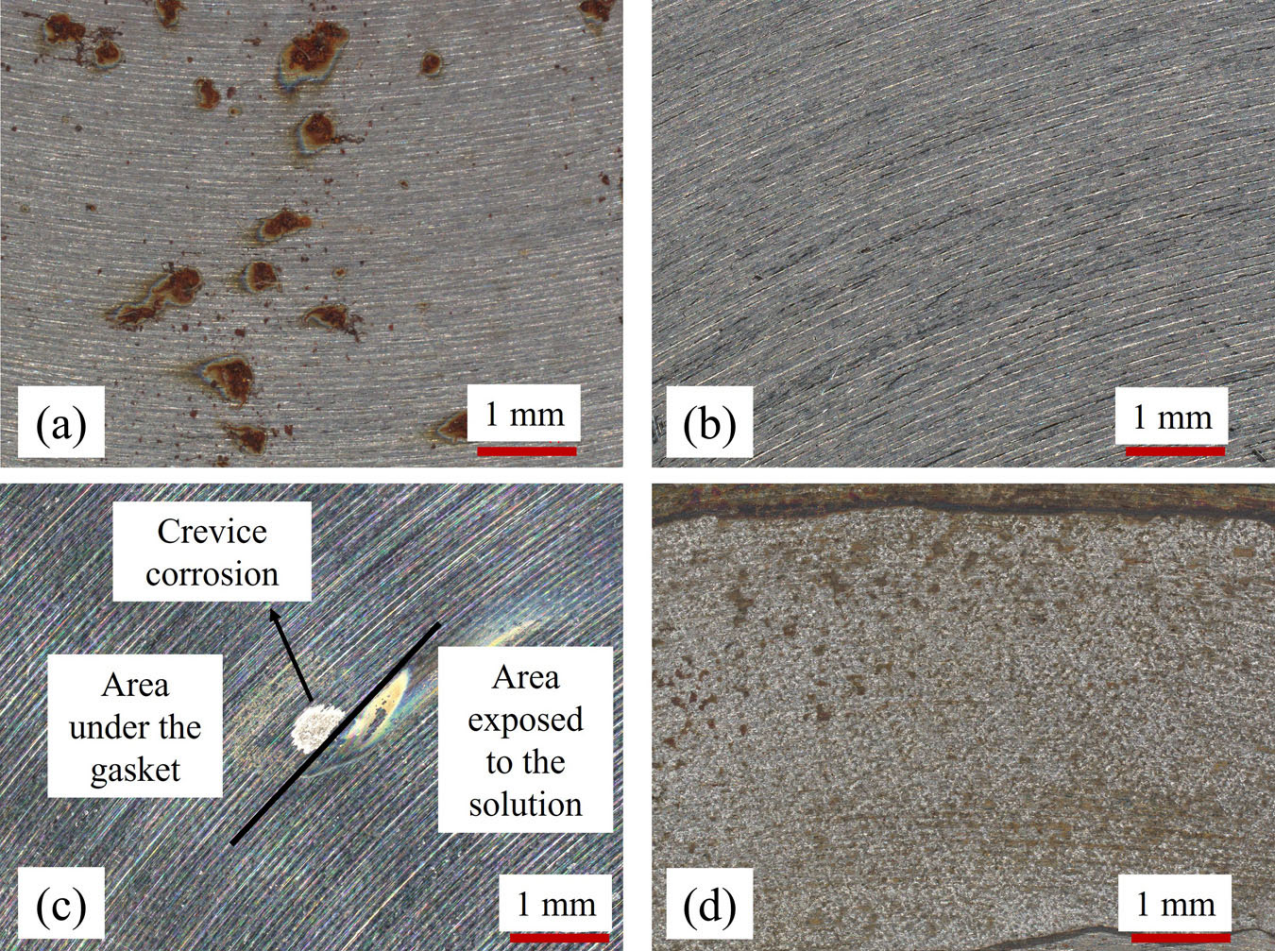
Micrographs of corroded surfaces. Microscopic images of the corroded areas on the flange sample plates, illustrating various types of corrosion after EN tests: (**a**) Pitting corrosion; (**b**) passive state; (**c**) crevice corrosion; (**d**) general corrosion.

Microscopic analysis of the flange sample plate surfaces after EN tests confirmed the presence of four distinct corrosion types on the flange sample plates. Figure 2a illustrates pitting corrosion, observed on plates that are passivated before exposure to the 0.5 M NaHCO_3_ + 0.1 M NaCl solution. Figure 2b shows a passivated flange sample with no visible signs of corrosion. In Fig. 2c, crevice corrosion morphology is evident at the interface between the gasket and flange, consistent with literature reports that crevice corrosion typically occurs in this area of flanged gasketed joints^42^. As shown in Fig. 2c, the boundary line between the area under the gasket and the area freely exposed to the solution, where crevice corrosion initiates and propagates. Figure 2d shows that general corrosion occurs uniformly across the flange sample plate surface.

### Supervised learning techniques

**Fig. 3:**
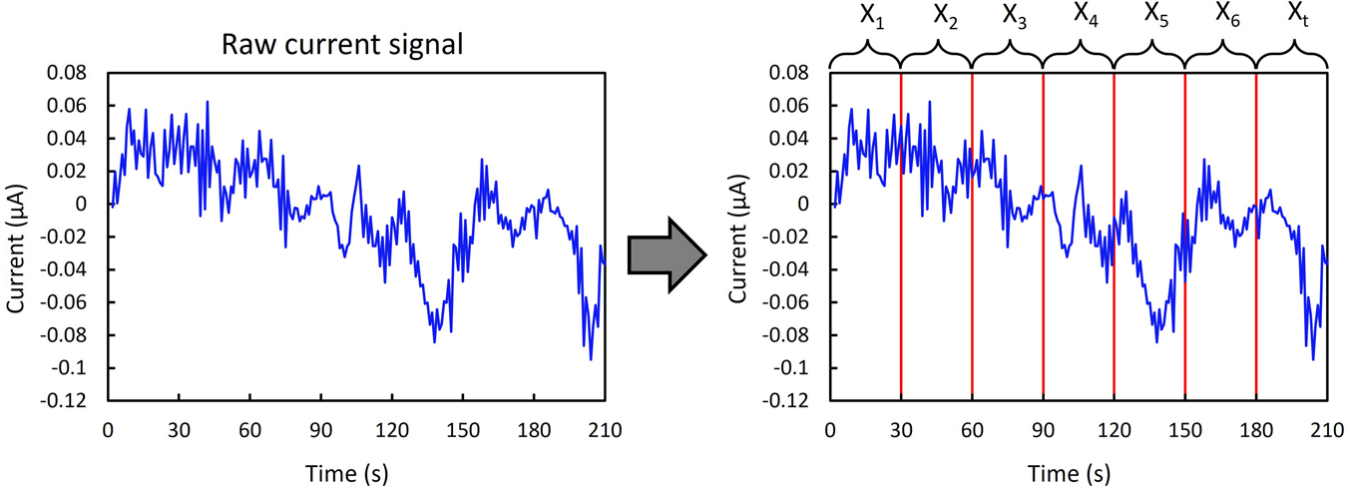
Sequencing of raw data. Example of the transformation of the raw current signal to the sequences of data that are used directly in the RNN models including LSTM, Simple RNN, and GRU.

Hyperparameter tuning for the RNN models focuses on optimizing three key parameters: the number of layers (num_layers), the number of neurons per layer (units), and the sequence length (seq_length). The sequence length in RNN models is determined based on the dependency length present in the data, with the optimal sequence length being the one that best captures the patterns within the signals. Figure 3 provides an example of a raw current signal and demonstrates how it is divided into sequences (*X*_*1*_ to *X*_*t*_) used by the RNN models.

To optimize the model configurations, Keras Tuner with Bayesian Optimization is employed. The optimal values obtain after tuning are then used to evaluate the models on the test dataset, with the results summarized in Table 2. For the RF model, the hyperparameter tuning targeted parameters including the number of trees in the forest (n_estimators), maximum tree depth (max_depth), minimum samples required for a split (min_samples_split), minimum samples required at a leaf node (min_samples_leaf), and whether to use bootstrapping (bootstrap). This tuning is performed using the GridSearchCV method from the sklearn.model_selection library, which automates the search for the optimal hyperparameters by exploring the specified parameter grid, using cross-validation to assess different combinations. Table 2 presents the search space and optimized hyperparameter values for each model, highlighting the effectiveness of the tuning approach in improving model performance.

**Table 2:** Hyperparameters, search spaces explored, optimised values, and best test accuracy for each model used for training

Model	Hyperparameters	Search space	Optimised value
LSTM	Sequence length (seq_length)	10 to 100 in steps of 10	30
	number of hidden layers (num_layers)	1 to 3	2
	number of units (units)	32 to 128	32, 64
Simple RNN	sequence length (seq_length)	10 to 100 in steps of 10	80
	number of hidden layers (num_layers)	1 to 3	2
	number of units (units)	32 to 128	96, 64
GRU	sequence length (seq_length)	10 to 100 in steps of 10	30
	number of hidden layers (num_layers)	1 to 3	2
	number of units (units)	32 to 128	128, 64
RF	n_estimators	10, 50, 100	50
	max_depth	None, 10, 20, 30	10
	min_samples_split	2, 5, 10	2
	min_samples_leaf	1, 2, 4	1
	bootstrap	True, False	False

**Fig. 4:**
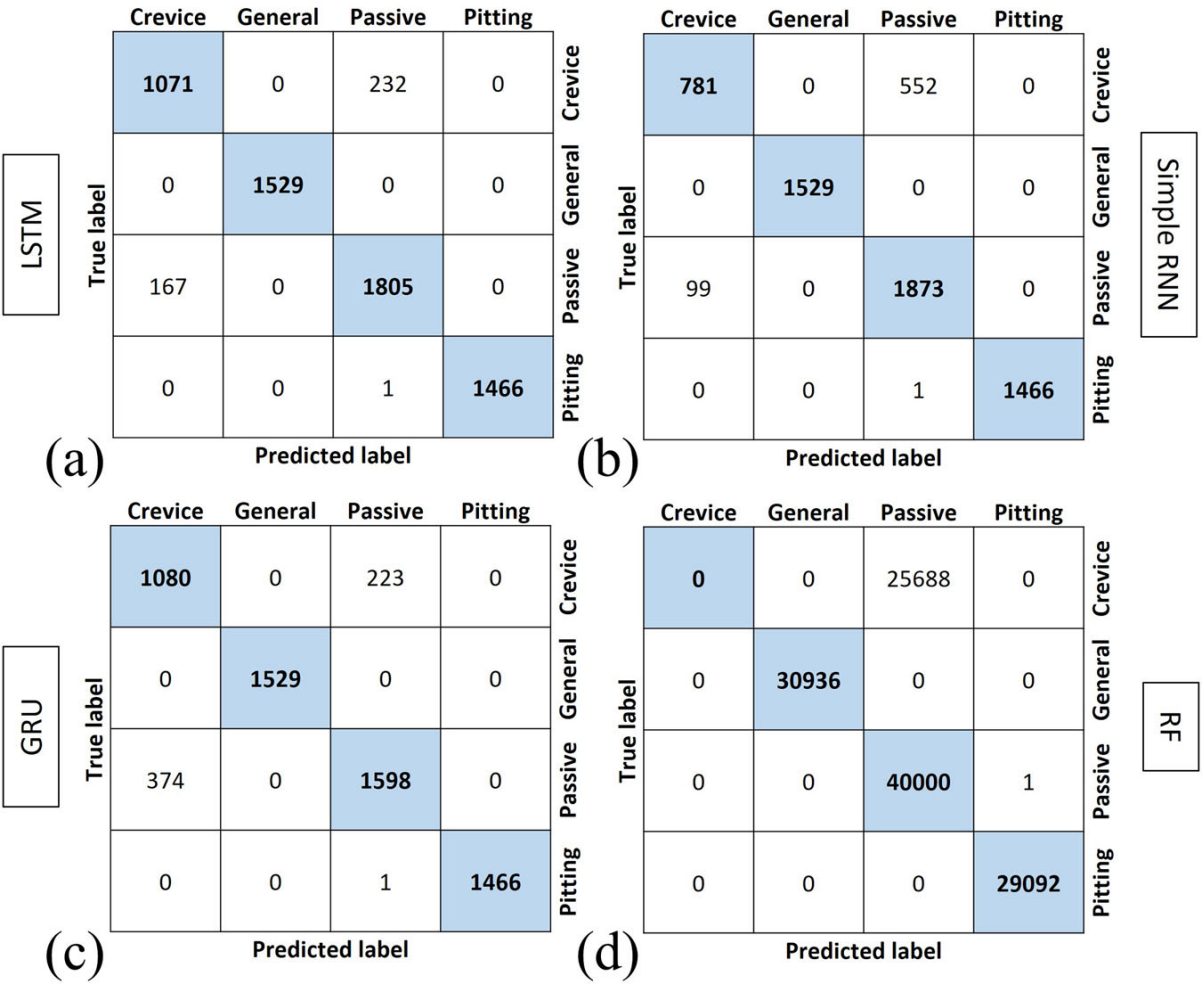
Confusion matrices for supervised models. Confusion matrices for the classification performance of models trained with optimized hyperparameters: (**a**) LSTM; (**b**) Simple RNN; (**c**) GRU; (**d**) RF.

Figure 4 shows the confusion matrices for all the trained models, and it indicates the performance of the models in classification and identification of the types of corrosion. The vertical axis in these images shows the True label of the test data and the horizontal axis shows the Predicted labels by the models. The diagonal of the confusion matrix shows the correctly detected types of corrosion. As shown in this figure, crevice corrosion is the most challenging type of corrosion to be detected. There is misidentification between crevice corrosion and passive state by all models but this misclassification is significantly observed with the RF model.

The LSTM model shows high accuracy for most corrosion types, with perfect classification for “General” and “Pitting” corrosion (1529 and 1466 correct predictions, respectively). There is, however, some misclassification of “Crevice” and “Passive” categories. Specifically, 232 instances that belong to the “Crevice” category are misclassified as “Passive,” while 167 “Passive” samples are identified as “Crevice.” These misclassifications suggest that the LSTM model struggles to differentiate between these two types of corrosion, potentially due to similarities in the EN signals during testing.

The Simple RNN model demonstrates lower performance compared to the LSTM model, particularly with the “Crevice” category, where 552 instances are misclassified as “Passive.” This model identifies a high number of FNs of crevice corrosion. Despite these issues, the Simple RNN model still performs well for general and pitting corrosion, with perfect classification for both categories.

The GRU model performance is relatively similar to LSTM, with slightly higher misclassifications of “Crevice” and “Passive” categories. For example, 374 “Passive” instances are classified as “Crevice,” indicating some overlap in how these two categories are interpreted by the model. The GRU model effectively identifies general and pitting corrosion without any misclassifications, except one instance of misclassification of pitting corrosion, suggesting its strength in handling distinct corrosion signals.

The RF model shows lower performance across all categories, with very high misclassification rates, especially for “Crevice” and “Passive” categories. For instance, nearly all “Crevice” instances (25,688) are misclassified as “Passive”. This indicates that the RF model struggles to capture the temporal dependencies in the data, which are crucial for distinguishing between different corrosion types. The inability of RF to handle sequential patterns as effectively as RNN-based models could be the primary reason for its poor performance. Overall, the RNN-based models (LSTM, Simple RNN, and GRU) outperform the RF model in classifying corrosion types.

Table 3 indicates an evaluation of the DL models and RF for classifying corrosion types using EN data. The performance metrics include precision, recall, F1-score, and best test accuracy for crevice corrosion, general corrosion, passive state, and pitting corrosion.

**Table 3:** Classification report showing precision, recall, and F1-score for different corrosion types using supervised learning models

Model	Type of corrosion	Precision	Recall	F1-score	Best test accuracy (%)
LSTM	Crevice	0.87	0.82	0.84	93.62
	General	1.00	1.00	1.00	
	Passive	0.89	0.92	0.90	
	Pitting	1.00	1.00	1.00	
Simple RNN	Crevice	0.89	0.60	0.72	90.08
	General	1.00	1.00	1.00	
	Passive	0.78	0.95	0.86	
	Pitting	1.00	1.00	1.00	
GRU	Crevice	0.74	0.83	0.78	90.46
	General	1.00	1.00	1.00	
	Passive	0.88	0.81	0.84	
	Pitting	1.00	1.00	1.00	
RF	Crevice	0.00	0.00	0.00	79.52
	General	1.00	1.00	1.00	
	Passive	0.61	1.00	0.76	
	Pitting	1.00	1.00	1.00	

The LSTM model exhibits strong performance across all corrosion types, with an overall best test accuracy of 93.62%. The LSTM’s performance is notable for general and pitting corrosion, achieving perfect precision, recall, and F1-scores (1.00), indicating that the model correctly identifies these corrosion types with no FPs or FNs. For the passive state, the model maintains high precision (0.89) and recall (0.92), resulting in a F1-score of 0.90. However, crevice corrosion shows lower metrics, with a precision of 0.87, recall of 0.82, and an F1-score of 0.84, reflecting some misclassification issues.

The Simple RNN model achieved a lower overall accuracy (90.08%) compared to LSTM. While it also performed perfectly on general and pitting corrosion (precision, recall, and F1-score of 1.00), the performance drops significantly for crevice corrosion, with an F1-score of 0.72. The precision-recall difference for crevice corrosion (0.89 precision vs. 0.60 recall) suggests that while the model can correctly identify some crevice cases, it struggles to detect all instances, leading to a higher rate of FNs. The performance on passive state (F1-score of 0.86) indicates that the simple RNN is effective in identifying this type of corrosion, although it lags behind the LSTM’s accuracy.

The GRU model shows performance with an overall accuracy of 90.46%. The results for general and pitting corrosion remain perfect (1.00 for all metrics), similar to the other models. However, for crevice corrosion, the GRU achieves an F1-score of 0.78, which is better than the Simple RNN but still lower than the LSTM performance. This suggests that the GRU ability to retain temporal dependencies helps to some extent, but the model may still struggle with distinguishing features of crevice corrosion. For the passive state, the GRU model shows slightly lower precision (0.88) compared to the LSTM, resulting in an F1-score of 0.84. This indicates that the GRU model, may not generalize as well as the LSTM for some corrosion types.

The RF classifier has the lowest overall test accuracy at 79.52%. While it performs perfectly on general and pitting corrosion, the metrics for crevice corrosion are poor, with precision, recall, and F1-scores all at 0.00. This indicates that the model fails to identify any instances of crevice corrosion, which could be due to the complexity of electrochemical noise data that requires capturing sequential dependencies, which cannot be achieved by the RF algorithm. For the passive state, the RF model achieves a recall of 1.00 but has a lower precision (0.61), leading to an F1-score of 0.76. This suggests that while the model is able to detect all instances of passive state, it also misclassifies other corrosion types as passive, resulting in a high number of FPs.

The LSTM model outperforms the others, achieving the highest overall accuracy and consistently high F1-scores across all corrosion types. All models struggle with accurately identifying crevice corrosion. This indicates that crevice corrosion may have features that overlap with other corrosion types, making classification difficult for non-sequential models like RF, or even simpler sequential models, such as Simple RNN. All models achieve perfect scores for general and pitting corrosion, suggesting that the distinguishing features for these corrosion categories are well-represented in the dataset. Although all models perform relatively well, there is still room for improvement in handling crevice corrosion and the passive state. Indeed, the results indicate that recurrent models are well-suited for analyzing EN data to classify different types of corrosion. The sequential nature of these models allows them to capture temporal dependencies in the data that traditional algorithms, such as RF cannot identify.

**Fig. 5:**
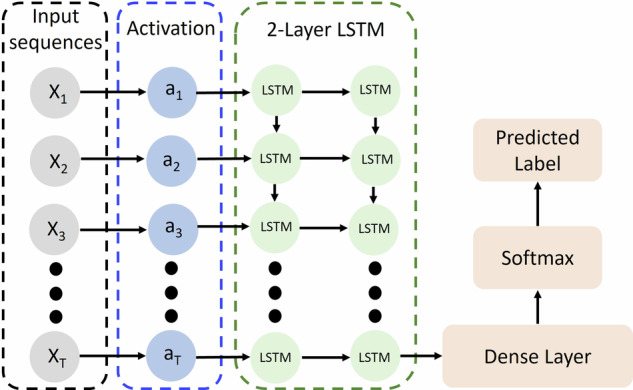
LSTM model structure. Structure of the tuned LSTM model with 2 layers and tanh as activation function.

As shown in Table 3, the LSTM model achieves the highest test accuracy among all the evaluated models. This performance is reached when using a sequence length of 30 and a two-layer LSTM architecture, as depicted in Fig. 5. The architecture includes 32 units in the first LSTM layer and 64 units in the second layer. The model input is a matrix of dimensions T × l, where l represents the length of the sequences and T denotes the number of sequences. Each input is first passed through a tanh activation function, which facilitates the non-linear transformation of the data before entering the LSTM layers. In this architecture, the final Dense layer, combined with a softmax activation function, is responsible for classifying the input into the target categories. This softmax layer outputs a probability distribution over the possible classes, and then the predicted label is the one that has the highest probability.

Compared with previous studies, which focus on extensive feature engineering to enhance classification, the present study demonstrates that RNN models - particularly LSTM - can perform well using only two input features: current and potential noise. While RF models in the literature have often required numerous input features to classify corrosion types, they typically showed lower performance and struggled with differentiating between passivation and pitting corrosion^43^. This limitation in previous RF models may come from the lack of hyperparameter tuning, which is addressed in this study, contributing to the improved performance of the RF model.

As discussed in this section, the RNN models perform well in differentiating various types of corrosion using EN data, surpassing the performance of the RF model. A key accomplishment of the developed supervised learning approach employed in this study is the use of only two input features, current and potential, in the RNN models. As highlighted in the introduction, it is common in the literature to engineer a large number of features derived from current and potential signals to classify different types of corrosion. However, the results of the present study indicate that only two features, current and potential, are sufficient for capturing the sequential dependencies and recurring patterns in EN data using RNN models. This leads to a significant reduction in computational costs, which is particularly important for real-world corrosion monitoring applications, where large datasets are typically generated.

Although the RF model shows lower performance compared to the RNN models, it detects all instances of general and pitting corrosion. This represents a notable improvement over previously reported results in the literature for identifying these corrosion types. The enhanced performance of the RF model in distinguishing general and pitting corrosion can be attributed to the hyperparameter tuning applied in this study, an aspect not extensively explored in prior research.

### Hybrid learning

As discussed in the literature review section, previous studies have employed various feature extraction techniques to generate predictors for ML models. In the previous section, it is demonstrated that RNN models can effectively classify different types of corrosion using labeled datasets and only two input features: the obtained current and potential signals by ENM. In this section, a hybrid approach combining supervised and unsupervised learning techniques is applied to train the RNN and RF models. The aim of this hybrid approach is to improve the classification performance of the models by automating the feature selection process through the use of an LSTM autoencoder. Hyperparameter tuning is employed to optimize the parameters of the LSTM autoencoder, and the resulting values are presented in Table 4.

**Table 4:** Hyperparameters, search spaces, and optimized values for the LSTM autoencoder model

Model	Hyperparameters	Search space	Optimized value
LSTM autoencoder	Sequence length (seq_length)	10–100	20
	number of hidden layers (num_layers)	1 to 3	2
	number of units (units)	32–128	50, 50

**Fig. 6:**
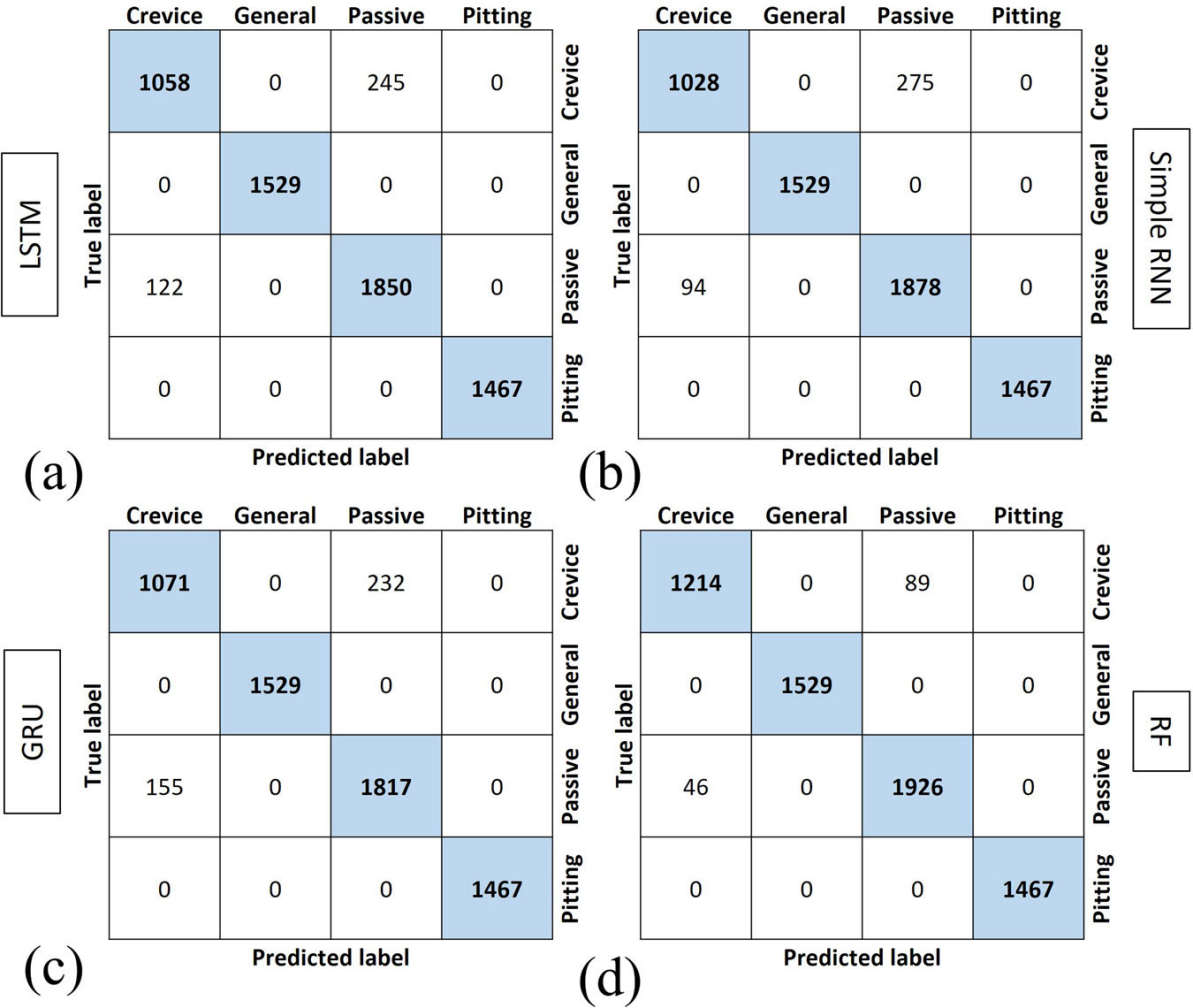
Confusion matrices for hybrid models. Confusion matrices obtained after training the hybrid model including the LSTM autoencoder and then supervised learning models (**a**) LSTM; (**b**) Simple RNN; (**c**) GRU; (**d**) RF.

The extracted features from the LSTM autoencoder are directly used in the supervised models discussed in the previous section. The LSTM autoencoder is an unsupervised DL technique which extracts the most important features from data without labeling the data. The confusion matrices obtained after training the models are shown in Fig. 6. The LSTM model exhibits robust performance across various corrosion types. For example, the model correctly classifies 1058 crevice corrosion instances, with a relatively small number of misclassifications (245 samples) categorized as “Passive.” Both general and pitting corrosion types are perfectly classified, which indicates that the LSTM model effectively handles these categories. In comparison, the Simple RNN model shows a slightly lower accuracy. While 1028 crevice corrosion instances are classified correctly, 275 crevice corrosion instances are misclassified as passive state, highlighting the model challenge in distinguishing between these two types of corrosion. This suggests that the LSTM model, with its memory retention capabilities, performs better than Simple RNN for temporal data patterns. However, similar to the LSTM model, the Simple RNN correctly classifies all general and pitting corrosion instances. The GRU model demonstrates robust performance, with 1071 correct classifications for crevice corrosion and fewer FPs (232) compared to the LSTM model. However, the GRU model has more FPs for passive state than the LSTM model. For general corrosion, all 1529 instances are classified correctly, and similarly, all 1466 pitting corrosion instances are accurately identified. This shows that while the GRU efficiently handles sequential data, the LSTM architecture slightly outperforms it in distinguishing between corrosion forms. The RF model displays a significant improvement in detecting crevice corrosion, with 1,214 correct classifications - higher than the other models - and only 89 FPs, which is lower compared to other models. Moreover, the RF model excels in detecting and differentiating the passive state from crevice corrosion, with 1926 correct classifications and only 46 misclassifications. Similar to the other models, RF correctly classifies all general and pitting corrosion instances. The performance improvement, particularly in the RF model, is attributed to the automatic feature extraction capability of the LSTM autoencoder, which selects the most critical features for training the subsequent supervised model.

Table 5 compares the performance of the four models - LSTM, Simple RNN, GRU, and RF - in identifying different types of corrosion, as indicated by precision, recall, F1-score, and test accuracy.

**Table 5:** Classification report showing precision, recall, and F1-score for different corrosion types using unsupervised LSTM autoencoder and supervised learning models

Model	Type of corrosion	Precision	Recall	F1-score	Best test accuracy (%)
LSTM	Crevice	0.9	0.81	0.85	94.15
	General	1.00	1.00	1.00	
	Passive	0.88	0.94	0.91	
	Pitting	1.00	1.00	1.00	
Simple RNN	Crevice	0.92	0.79	0.85	94.12
	General	1.00	1.00	1.00	
	Passive	0.87	0.95	0.91	
	Pitting	1.00	1.00	1.00	
GRU	Crevice	0.87	0.82	0.85	93.83
	General	1.00	1.00	1.00	
	Passive	0.89	0.92	0.90	
	Pitting	1.00	1.00	1.00	
RF	Crevice	0.96	0.93	0.95	97.85
	General	1.00	1.00	1.00	
	Passive	0.96	0.98	0.97	
	Pitting	1.00	1.00	1.00	

LSTM shows solid performance for crevice corrosion detection with an F1-score of 0.85, though it slightly underperforms in recall (0.81), indicating that the model occasionally misses some crevice corrosion instances. The LSTM model achieves a perfect score (Precision, Recall, and F1-score of 1.00) for both general and pitting corrosion. This suggests that the LSTM captures the characteristics of these corrosion types. This model achieves an F1-score of 0.91 for the passive state, reflecting a well-balanced performance. Its recall is higher than precision (0.94 vs. 0.88), showing that while it correctly identifies most passive state cases, a few FPs are included. With a best test accuracy of 94.15%, the LSTM model is highly reliable overall, particularly for identifying corrosion types like pitting and general corrosion.

The Simple RNN achieves similar results as the LSTM for crevice corrosion, with an F1-score of 0.85 and slightly higher precision (0.92), which indicates better identification of crevice corrosion instances compared to LSTM. Like the LSTM, Simple RNN achieves perfect scores (1.00) in both general and pitting corrosion, demonstrating the model ability to handle clear and distinct patterns in these types of corrosion. The model achieves an F1-score of 0.91 for passive state, similar to LSTM, but it has slightly better recall (0.95 vs. 0.87 precision). This suggests that the model excels at capturing true passive state cases, though it might include some misclassifications. The Simple RNN achieves an overall test accuracy of 94.12%, which is roughly equal to the test accuracy of LSTM model. Its performance on crevice corrosion is notable, as it demonstrates higher precision than LSTM.

The GRU model has an F1-score of 0.85 for crevice corrosion, with balanced precision (0.87) and recall (0.82). This is slightly below the performance of both LSTM and Simple RNN but remains a good result overall. Like the other RNN-based models, the GRU achieves perfect scores (1.00) for general and pitting corrosion, suggesting that it can handle clearly distinguishable corrosion patterns well. With an F1-score of 0.90, GRU performs slightly below the LSTM for passive state but still exhibits a strong balance between precision (0.89) and recall (0.92). With a best test accuracy of 93.83%, the GRU model performs slightly below the LSTM and Simple RNN models but remains a competitive option. Its performance on all corrosion types is strong, though it appears to face similar challenges in differentiating crevice corrosion and passive state.

The RF model excels in detecting crevice corrosion, achieving an F1-score of 0.95 with a high recall (0.93) and precision (0.96). This indicates a superior ability to correctly identify and classify crevice corrosion compared to the RNN models. Like the RNN models, RF achieves perfect scores (1.00) for general and pitting corrosion, meaning it effectively handles these corrosion types. RF demonstrates outstanding performance for passive state detection, with an F1-score of 0.97. Its recall (0.98) is higher than precision (0.96), meaning that while it detects almost all instances of passive state, it may occasionally misclassify other types as passive. With a best test accuracy of 97.85%, the RF model outperforms the RNN-based models in terms of overall accuracy. This indicates that RF is particularly robust when trained on features extracted by the LSTM autoencoder and can differentiate between corrosion types more effectively than the sequential models.

**Fig. 7:**
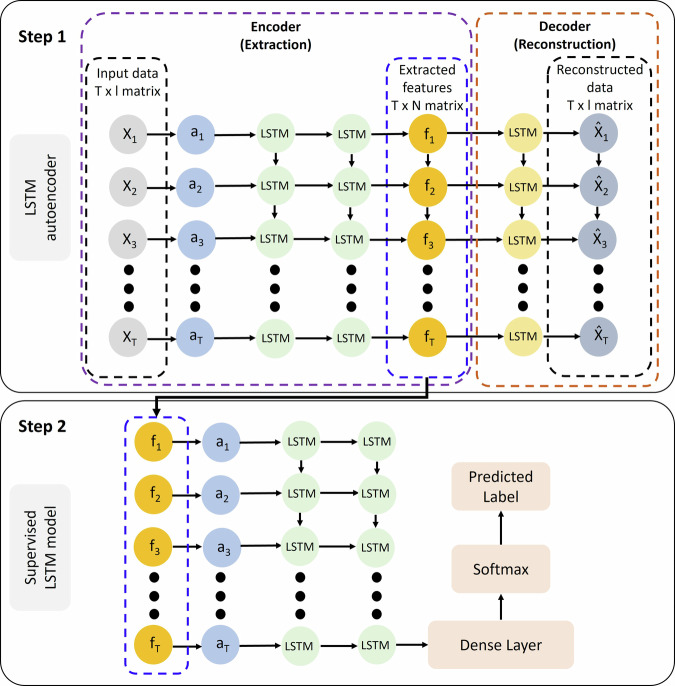
Autoencoder and LSTM model structure. Architecture of the hybrid approach, incorporating feature extraction using a two-layer LSTM autoencoder (unsupervised technique) with 50 units per layer, followed by classification using a two-layer LSTM model (supervised technique) with 32 and 64 units.

The structure of the hybrid model is illustrated in Fig. 7. The input data is first passed through the LSTM autoencoder, where critical features are automatically extracted from the raw electrochemical current and potential signals during the encoding phase. These extracted features form a matrix of dimensions T × N, where T represents the number of sequences and N denotes the number of extracted features. In the subsequent decoding step, the LSTM autoencoder attempts to reconstruct the input data from the extracted features, and the reconstructed data is compared with the original input to assess the autoencoder’s performance in capturing essential features. The LSTM autoencoder architecture consists of two LSTM layers with tanh activation for encoding and one LSTM layer for decoding. The latent representations generated by the LSTM autoencoder are then fed into DL and ML models for classification of different corrosion types. For simplicity, Fig. 7 only illustrates the LSTM model, though other DL models are used as well. The first step of the hybrid model is unsupervised, as the LSTM autoencoder evaluates the extracted features by reconstructing the input data without the need for labeled data. The second step is a supervised learning process, in which labeled datasets are used to classify the corrosion types through DL and ML models.

The hybrid learning approach, underscores the LSTM autoencoder ability to capture complex patterns and dependencies of EN data, which enhances the classification accuracy of both RNN-based models and, particularly, the RF model. The RF model, traditionally less effective in handling sequential data, benefits from the autoencoder learned features, which retain key temporal dependencies. Consequently, the LSTM autoencoder proves to be a powerful tool in reducing the need for extensive feature engineering, allowing the models to focus on essential patterns within current and potential noise as input features, thereby improving classification accuracy and efficiency in corrosion monitoring applications.

### Unsupervised learning

Since real-world corrosion monitoring scenarios often involve obtaining unlabeled data from EN tests, an unsupervised learning technique to differentiate between various types of corrosion on flange surfaces using unlabeled data is finally proposed. This approach consists of two main steps. In the first step, critical features are automatically extracted using an LSTM autoencoder, and in the second step, these features are used as inputs for clustering via the k-means algorithm. The hyperparameters utilized in the LSTM autoencoder are shown in Table 6 and are optimized within a defined search space. Two sequence lengths, 20 and 700, are tested with this approach to evaluate the model differentiation performance across different types of corrosion, as variations in sequence length affect the model classification capabilities for specific corrosion types.

**Table 6:** Hyperparameters, search spaces, and optimized values for the LSTM autoencoder model for unsupervised learning

Model	Hyperparameters	Search space	Optimized value
LSTM autoencoder	Sequence length (seq_length)	10–1500	20, 700
	number of hidden layers (num_layers)	1 to 3	2
	number of units (units)	32 to 128	50, 50

**Fig. 8:**
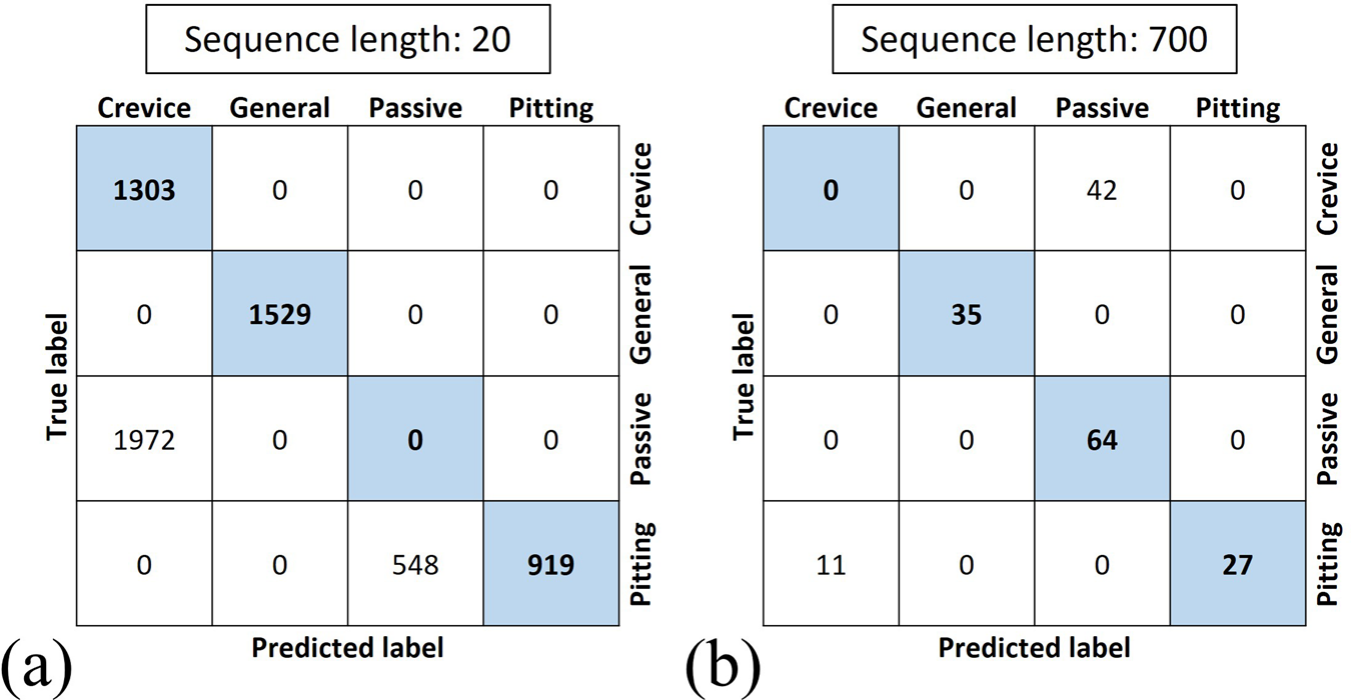
Confusion matrices for unsupervised models. Confusion matrices obtained after training the LSTM autoencoder and then k-means algorithm in the (**a**) sequence length of 20; (**b**) sequence length of 700.

Figure 8 presents the confusion matrices for the hybrid learning technique at two different sequence lengths. In Fig. 8a, which corresponds to a sequence length of 20, the model successfully distinguishes all instances of crevice corrosion, demonstrating complete accuracy in identifying this type of corrosion. Similarly, general corrosion cases are entirely classified correctly. However, the model misclassifies all instances of the passive state as crevice corrosion. For pitting corrosion, the model accurately identifies 919 instances, but 548 instances are erroneously categorized as the passive state. Thus, with a sequence length of 20, the hybrid model effectively differentiates crevice and general corrosion, though it struggles with the passive state and pitting corrosion. In contrast, when the sequence length is increased to 700, as shown in Fig. 8b, the model exhibits an improved performance for all corrosion types except crevice corrosion. In this case, all instances of crevice corrosion are misclassified as the passive state. Despite this limitation, the model correctly classifies all cases of general corrosion and the passive state. For pitting corrosion, 27 cases are accurately identified, while 11 cases are misclassified as crevice corrosion. This comparison indicates that, while a sequence length of 700 enhances the model ability to differentiate most corrosion types, it introduces challenges in correctly identifying crevice corrosion.

The evaluation metrics for measuring model performance are presented in Table 7. For a sequence length of 20, the model achieves a precision of 0.40, a recall of 1.00, and an F1-score of 0.57 for detecting crevice corrosion. In contrast, for general corrosion, all three metrics—precision, recall, and F1-score—are 1.00, indicating perfect classification performance. The model performance for the passive state is notably poor, with all metrics recorded as 0.00. For pitting corrosion, the model achieves a precision of 1.00, a recall of 0.71, and an F1-score of 0.83. The overall test accuracy of the hybrid model for a sequence length of 20 is 59.82%. When using a sequence length of 700, the model performance changes notably. For crevice corrosion, all evaluation metrics are 0.00, indicating a complete misclassification. For general corrosion, all metrics remain at 1.00, showing consistent accuracy. For the passive state, the model achieves a precision of 0.60, a recall of 1.00, and an F1-score of 0.75. For pitting corrosion, the precision remains at 1.00, while recall is 0.71, and the F1-score is 0.83. The highest test accuracy observed for the model with a sequence length of 700 is 70.39%.

**Table 7:** Classification report showing precision, recall, and F1-score for different corrosion types using unsupervised LSTM autoencoder with clustering in different sequence lengths

Sequence length	Model	Type of corrosion	Precision	Recall	F1-score	Best test accuracy (%)
20	LSTM autoencoder with k-means	Crevice	0.40	1.00	0.57	59.82
		General	1.00	1.00	1.00	
		Passive	0.00	0.00	0.00	
		Pitting	1.00	0.63	0.77	
700	LSTM autoencoder with k-means	Crevice	0.00	0.00	0.00	70.39
		General	1.00	1.00	1.00	
		Passive	0.60	1.00	0.75	
		Pitting	1.00	0.71	0.83	

**Fig. 9:**
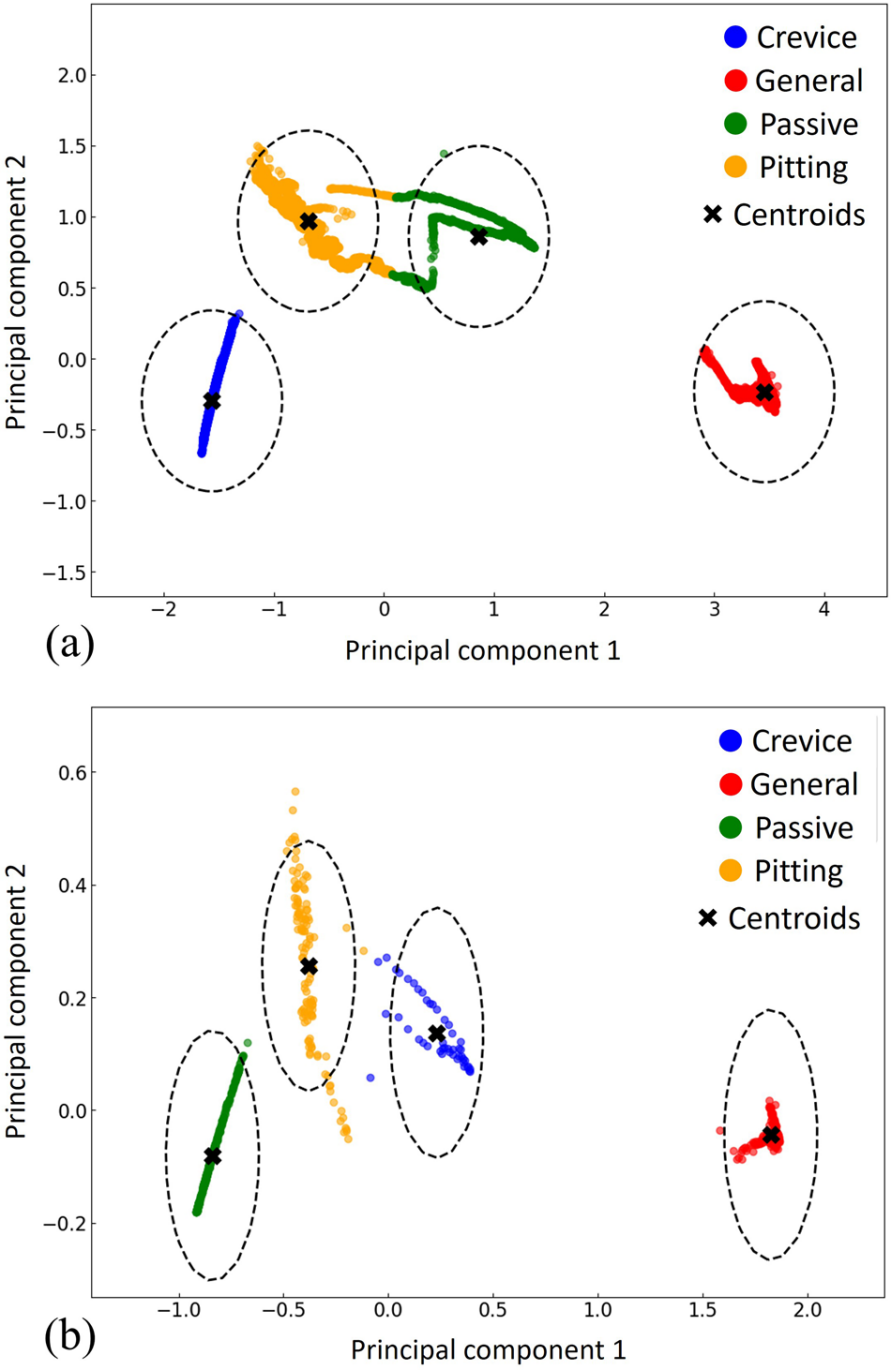
PCA visualization of unsupervised models. Visualization of the extracted features from the LSTM autoencoders using PCA method in two dimensions for the (**a**) sequence length of 20; (**b**) sequence length of 700.

The PCA visualization of the encoded features illustrates the separation between different types of corrosion in the latent space. In Fig. 9, each color in the scatter plot corresponds to a specific type of corrosion: crevice, general, passive state, and pitting. PCA is used here to project the high-dimensional latent features extracted by the LSTM autoencoder into two principal components, making it easier to visualize the distinction of corrosion types. As shown in Fig. 9a, which is related to the sequence length of 20, the clusters for crevice corrosion (in blue) and general corrosion (in red) are distinctly isolated from the other types. This suggests that, on the one hand, the encoded features corresponding to these corrosion types are unique enough to be reliably distinguished. On the other hand, there appears to be a slight overlap between the clusters for pitting (in yellow) and passive states (in green). This overlap could indicate some similarities in the features between these two corrosion types.

In Fig. 9b, which corresponds to a sequence length of 700, the general corrosion and passive states are clearly distinguished from the other corrosion types. This clear isolation indicates that the extracted features effectively capture the differences between these types and the rest. However, there is a slight overlap between the clusters representing pitting and crevice corrosion, suggesting that the extracted features from inputs with a sequence length of 700 learned the dependencies in the data more effectively for distinguishing general corrosion and passive states.

After transforming the extracted features into a lower-dimensional space using PCA, k-means clustering is used to group similar data points, where each cluster represents a specific corrosion type. Within each cluster, the distance of each point to its centroid is calculated. The centroid, identified by a black cross in Fig. 9 represents the typical behavior for a given corrosion type. The 95% threshold or control limit is set based on the 95^th^ percentile of these distances (shown by a black dashed line in Fig. 9a, b), defining a boundary within which data is considered to be typical for the corrosion type associated with the cluster.

The unsupervised approach can be applied in real-time corrosion monitoring. For example, if the normal operating condition is general corrosion or passive state, the measured EN data will be located inside the control limits (dashed lines in Fig. 13) of these types of corrosion. If the input data is located outside of the control limits of general corrosion or passive state, it can be concluded that crevice corrosion or pitting corrosion is initiated in the flanged joint.

Although confusion matrices and classification metrics show lower performance for the unsupervised approach than the supervised and hybrid approaches, after applying PCA the types of corrosion could be distinctly identified and then clustered using k-means, specifically in the sequence length of 700 and for general and passive corrosion. Unsupervised approach has higher applicability in real-time corrosion monitoring than the other proposed approaches in this study as the corrosion monitoring data are mostly unlabeled.

To sum up, this study demonstrates the potential of RNN models including simple RNN, LSTM, GRU and particularly LSTM networks and autoencoders in distinguishing the types of corrosion by analyzing EN data obtained from flange sample plate surfaces under different experimental conditions. The findings and analyses reveal that:

- Among the supervised models, the LSTM achieved the highest test accuracy of 93.62%, effectively uncovering hidden patterns in the EN data, which enabled robust classification of corrosion types.
- To enhance the models’ accuracies, a hybrid approach is implemented, resulting in improved performance across all models. The RF model achieved the highest test accuracy of 97.85% in distinguishing corrosion types, demonstrating the effectiveness of feature extraction through LSTM autoencoders for pattern recognition.
- The supervised and hybrid approaches, leveraging labeled data, successfully distinguish between general corrosion, pitting, crevice corrosion, and passive states. However, the performance of the unsupervised technique, which operates without labeled data—a more typical scenario in real-world corrosion monitoring—is less effective in comparison.
- In the unsupervised approach, PCA assists in clustering based on features extracted by the LSTM autoencoder, improving its ability to detect transitions between corrosion types. In real-time monitoring scenarios, this system can continuously classify incoming EN data and detect shifts from passive states to aggressive forms of corrosion, such as pitting or crevice corrosion, based on the cluster assignments.

This study is the first in the literature that proposes the use of RNN models for processing EN data in corrosion monitoring. It was demonstrated that using the developed RNN approach the identification of localized corrosion initiation (pitting or crevice) can be automated, without the need for disassembly of bolted joints in pipelines that causes shutdowns and significant losses.

In future research, the developed approaches can be improved by adding more corrosion types to the database and increasing the range and type of service conditions. Then, the presented RNN-based model can be applied in corrosion monitoring to reassess the effectiveness of coatings and inhibitors, as data from ineffective coatings or inhibitors will be mapped to distinct clusters, enabling early detection of reduced performance. Using databases related to coatings and inhibitors, the model’s ability for detecting and evaluating the effectiveness of these protective measures can be validated and enhanced, and as such create a novel powerful tool for enhanced corrosion management and predictive maintenance in industrial environments. Furthermore, since the approach developed in this study is designed for use with raw EN signals without relying on system-specific features, it holds promise for generalization across various corrosion systems, including different materials and environments. Future validation studies could focus on applying this approach to datasets collected from marine, atmospheric, or sour service conditions to further demonstrate its adaptability to real-world use cases.

## Methods

**Fig. 10:**
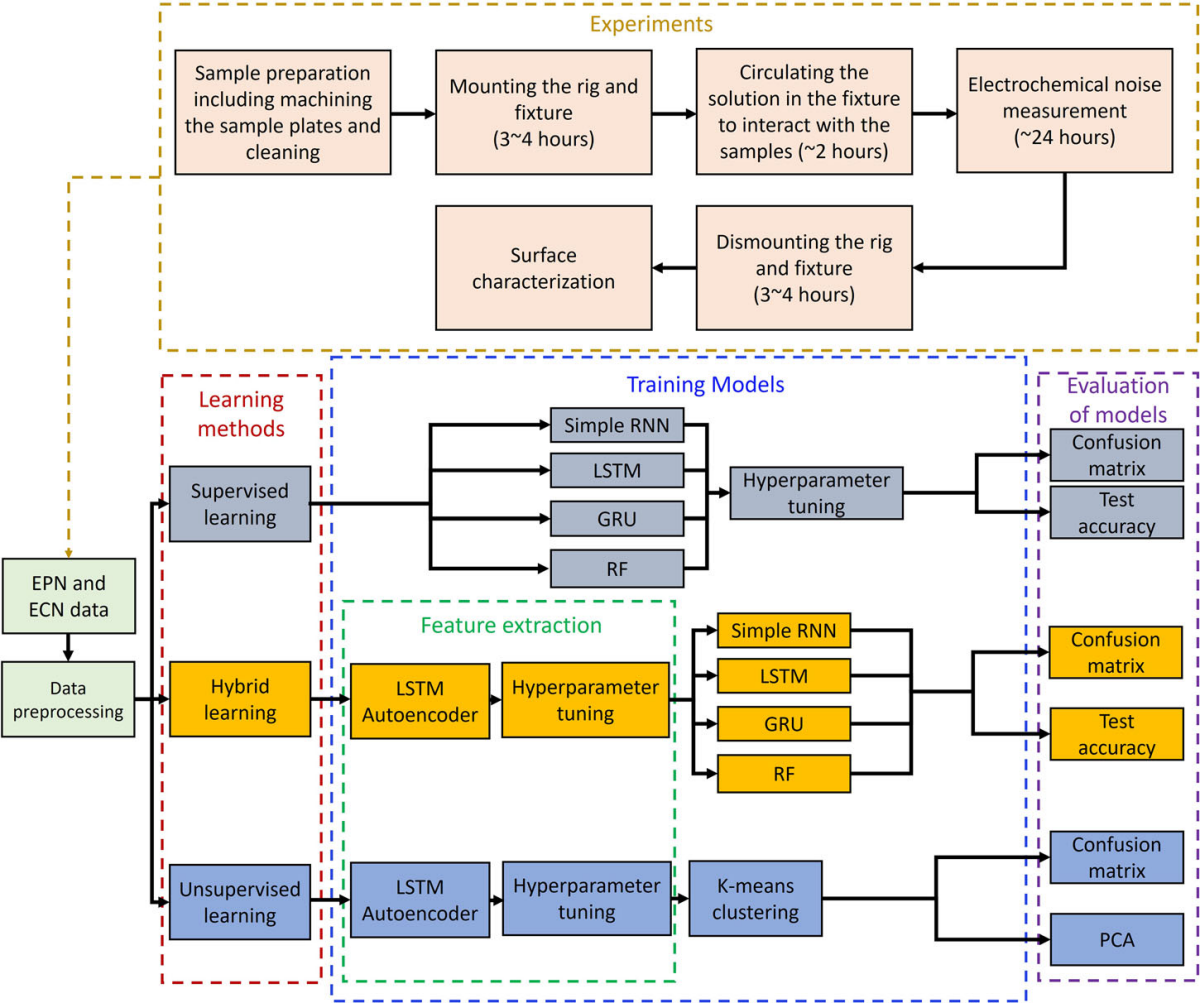
Methodology overview. Schematic overview of the methodology used for the classification of the type of corrosion.

The overview of the methodology used in this study is shown in Fig. 10. In order to study the applicability of RNN models to process EN data, in a first step, experimental tests are performed to collect data for model training. Then, collected data are preprocessed and prepared by removing outliers, labeling the dataset, and encoding categorical data, to feed the models. Subsequently, different learning models, as shown in Fig. 10, are trained and their performances are evaluated and compared with each other, using confusion matrices and other typical DL and ML performance metrics. Three approaches are considered, namely supervised learning, hybrid learning, and unsupervised learning. The supervised and hybrid learning models need labeled data to train and predict labels, but the unsupervised learning models are used in cases where data are not labeled, which is typically the case in uncontrolled, real-world environments. Hyperparameter tuning is conducted for each model to identify the parameter values that yield the highest accuracy. Confusion matrices are also used in the evaluation step to visualize the predicted corrosion types versus true corrosion types. All ML models are built using Python in Jupyter notebook. The details of each step in Fig. 10 is discussed in the following sections.

### Materials

**Fig. 11:**
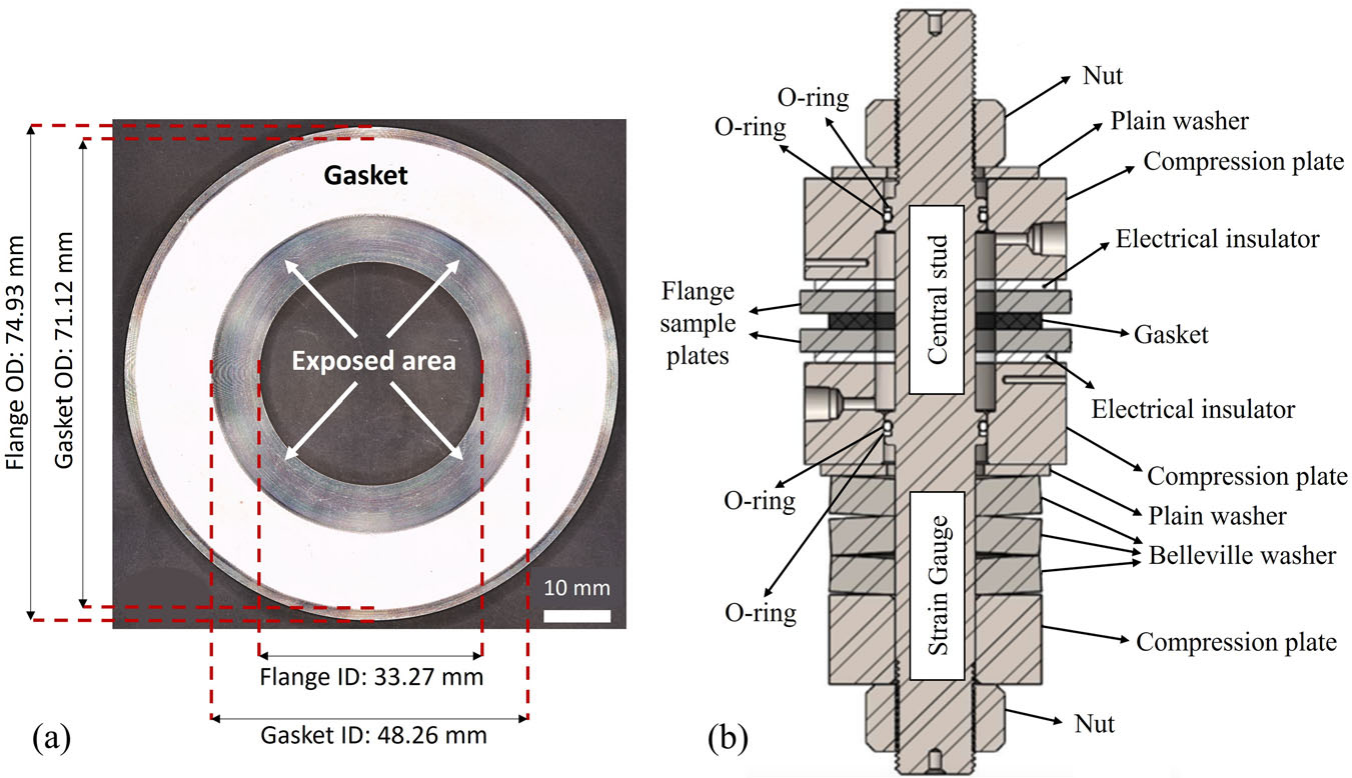
Illustration of flange, gasket, and fixture. The schematic illustration of the (**a**) flange sample plate including the sizes and the exposed area to the solution; and (**b**) the test fixture including the labels of each item in the fixture.

The materials of the sample plates are ASTM A105 carbon steel, and ASTM A182 F321 stainless steel (SS) which are widely used in the manufacturing of flanges. The chemical compositions of the flange materials are provided in Table 8. The flange sample plates have an outside diameter OD of 2.95 in. (74.93 mm), an inside diameter ID of 1.31 in. (33.27 mm), and a thickness of 0.25 in. (6.35 mm) (as shown in Fig. 11a). Virgin polytetrafluoroethylene (PTFE) gaskets are used between the sample plates, following the specifications of ASME B16.21^44^ for non-metallic flat gaskets used in flanges. The thickness of the gasket is 3.17 mm with the ID and OD of 48.26 and 71.12 mm, respectively (as shown in Fig. 11a). The surface area of the flange that is exposed to the solution is equal to 9.73 cm^2^ for each sample plate. The roughness of the sample plates is measured using a Mitutoyo Surftest SJ-410 mechanical profilometer following the ISO 21920-2:2021 standard, as commonly used in the literature^45^. A cut-off length of 0.8 mm and a short wavelength cut-off filter λ_s_ of 2.5 µm are used, resulting in an arithmetic mean of absolute height values Ra= 1.006 ± 0.05 µm after three measurements on three different samples.

**Table 8:** Chemical composition of flange sample plates (wt. %)

Elements	C	N	Si	P	S	Cr	Mn	Ni	Mo	Cu
321 SS	0.049	0.024	0.54	0.03	0.001	17.45	1.57	9	0.37	0.48
A 105	0.19	0.01	0.22	0.01	0.02	0.17	1.09	0.09	0.03	0.24

### Electrochemical tests

In order to perform electrochemical tests in conditions close to real-world flanged gasketed joints, the gasket is sandwiched between two flange sample plates as shown in Fig. 11a, and then placed in the fixture (Fig. 11b) of an in-house developed test rig^10,11^. Since flanged gasketed joints are secured using hydraulic tensioners that apply high initial compressive stress^46^, the fixture illustrated in Fig. 11b is positioned on a stand with a hydraulic tensioner to compress the gasket to an initial average stress level of 15 MPa. This contact stress is calculated based on the measurement of the central bolt force using a full Wheatstone bridge with strain gauges attached to the central stud before testing.

The fixture is composed of nuts for fastening the joints after applying the compressive load, plain washers to increase the contact area, compression plates that have entrance and exit ports for the solution, electrical insulators to avoid electrical short circuits between the flange sample plates, which are also used as working electrodes (W.E.1 and W.E.2), compression plates and Belleville washers to maintain the preload during the electrochemical tests. The O-rings are placed between the central stud and the compression plates to seal the solution chamber and avoid electrical short circuits between the central stud and the compression plates.

**Fig. 12:**
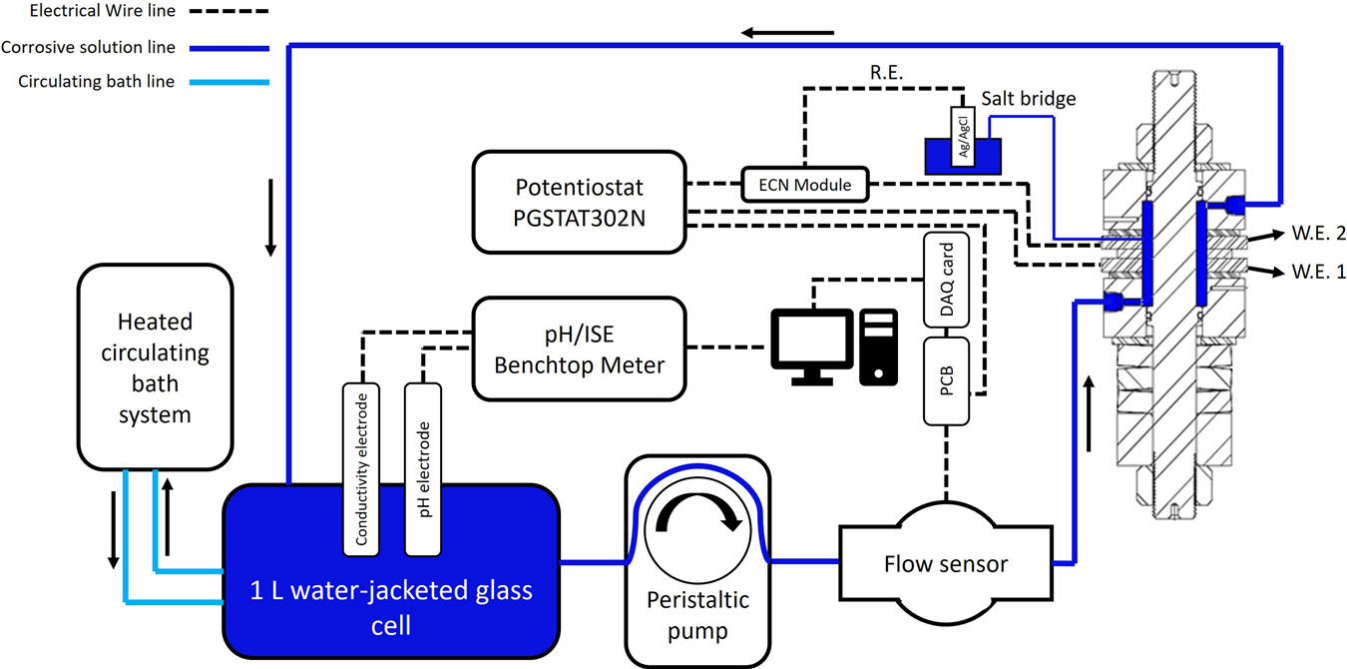
Test rig illustration. The schematic of the test rig including all the sensors and equipment for measurements and monitoring.

After mounting the fixture, the tubes and electric wires are connected as shown in Fig. 12. The electrolytic solution passes through a water-jacketed glass cell used to control and maintain the temperature to ±1 ^o^C. The water jacket surrounds the solution inside the glass cell and acts as a temperature buffer. The heated circulating bath system (Polystat Cole-Parmer CR500WU) controls and maintains the temperature of the water in the jacket side of the glass cell by a heating and cooling system. The electrolyte solution in the glass cell flows into the tubes (identified by the dark blue lines in Fig. 12) through the peristaltic pump (BRL Life Technologies CP-600). The solution flow rate is adjusted by the peristatic pump and measured by the flow sensor (Digiten FL-402B). The conductivity, pH, and temperature of the solution are measured by the conductivity and pH electrodes connected to a benchtop multiparameter meter (Thermo Fisher STARA2150 series). For the EN tests, a Metrohm Autolab PGSTAT302N High-Performance potentiostat/galvanostat, including a dedicated ECN module (Metrohm ECN.S X19-6), is employed to capture both current and potential data. A Pine Research single-junction, saturated Ag/AgCl reference electrode, equipped with a porous ceramic tip and filled with a 3 M KCl solution, serves as the reference electrode (R.E.), and all potentials are measured relative to this Ag/AgCl electrode. To minimize the effect of the ohmic drop between the reference and working electrodes, a salt bridge is used to connect the test solution in the fixture to the reference electrode. Sensor-generated analog signals are transmitted to a custom-designed printed circuit board (PCB) and digitized by a National Instruments data acquisition (DAQ) card. The DAQ, potentiostat, and multiparameter meter interface directly with the computer via USB, managed through a LabVIEW program.

The EN data are collected from four different experimental conditions (C1-C4). Hence, four test solutions are prepared using the analytical grades which are 0.1 M sodium chloride (NaCl) (C1), 0.5 M sodium hydrogen carbonate (NaHCO_3_) (C2), 0.45 M sodium hydrogen carbonate + 0.1 M sodium chloride (0.45 M NaHCO_3_ + 0.1 M NaCl) (C3), and 0.6 M sodium chloride (NaCl) (C4). These solutions are used to induce general corrosion, passivation, pitting, and crevice corrosion, respectively. To induce pitting corrosion, the sample plates are passivated in the 0.5 M NaHCO_3_ solution for 1 h before placing in the fixture for testing. The EN measurements are performed by connecting the upper flange sample plate as W.E. 1 and the lower one as W.E. 2 (as shown in Fig. 11b) in the test rig, which are nominally identical samples and parallel to each other.

The current between the two electrodes is measured using the ZRA mode of the Autolab potentiostat, and the potential of the W.E.s is measured relative to the R.E. using the high-resolution Metrohm ECN module. The EN data is collected with a frequency of 2 Hz. Table 9 indicates the experimental conditions to build the dataset for training and testing the classification ability of RNN models.

**Table 9:** Experimental conditions to make a dataset to test the classification ability of the RNN model

Condition	Rows of data	Material	Solution	Temperature (^o^C)	Type of corrosion	Time (h)
C1	154817	Carbon steel A105	0.1 M NaCl	22	General corrosion	21
C2	172712	Carbon steel A105	0.5 M NaHCO_3_	22	Passive	24
C3	144493	Carbon steel A105	0.5 M NaHCO_3_ + 0.1 M NaCl	22	Pitting corrosion	20
C4	53742	321 SS	0.6 M NaCl	50	Passive	7.5
C4	101130	321 SS	0.6 M NaCl	50	Crevice corrosion	14

The sample plates are degreased in an ultrasonic bath with ethanol for 20 min, followed by air drying before subjected to EN testing. The EN tests start two hours after letting the electrolyte solution circulate within the fixture, ensuring sufficient time for the surfaces of the sample plates and the interface with the gasket to soak. Each EN test is replicated three times to verify repeatability and reproducibility of the corrosion type occurring on the flange faces. The corrosion type is confirmed during post-test microscopic examination. However, only one representative dataset per condition is used in the model to avoid over-representation of similar signals and reduce the risk of overfitting.

### Flange surface analysis

Following each experiment, the flange sample plates are first rinsed with distilled water, then further cleaned with ethanol. The samples are subsequently air-dried at room temperature. The corroded surfaces are observed using a digital microscope (Keyence VHX-7000) with a VHX E20 lens with the tilt angle of 0 degree to characterize and determine the type of corrosion that took place on them.

### Data preprocessing

The potential and current signals obtained from the EN tests are labeled during the data preprocessing stage. These labels correspond to the type of corrosion observed in the signals and microscopic images: “General,” “Passive,” “Pitting,” and “Crevice.” The number of data entries associated with each corrosion type is as follows: “General” = 154,817; “Passive” = 226,454; “Pitting” = 144,493; and “Crevice” = 101,130, as detailed in Table 3. The categorical labels are then converted into numerical values using the LabelEncoder from the sklearn.preprocessing^47^ module, allowing the models to process the data. Linear detrending was applied to the current signal using scipy.signal.detrend^48^ to remove baseline offsets and slow drifts.

### Recurrent Neural Network (RNN)

RNNs are a type of neural network architecture featuring recurrent connections, primarily used to identify patterns within sequential data. This data can include handwriting, genetic sequences, speech, or numerical time series, commonly generated in industrial settings (e.g., by sensors)^49^. RNNs contain high-dimensional hidden states characterized by non-linear dynamics. This hidden state structure acts as memory for the network, with each hidden layer state at a given moment influenced by its preceding state^50^. This allows the network to maintain and update contextual information as it processes a sequence of data. The hidden state update is represented as Eq. 1, where *h*_*t*_ is the current hidden state, *h*_*(t-1)*_ is the previous hidden state, *x*_*t*_ is the current input, *W*_*h*_ and *W*_*x*_ are weight matrices, *b* is a bias term, and *f* is an activation function^51^. The output *y*_*t*_ of the RNN network is obtained by Eq. 2 at each time step *t*. The size of the hidden state is a hyperparameter that can be tuned. Larger hidden states can potentially capture more information but also require more computational resources.

1 $${{\rm{h}}}_{{\rm{t}}}={\rm{f}}({{\rm{W}}}_{{\rm{h}}}\times {{\rm{h}}}_{{\rm{t}}-1}+{{\rm{W}}}_{{\rm{x}}}\times {{\rm{x}}}_{{\rm{t}}}+{{\rm{b}}}_{{\rm{h}}})$$

2 $${{\rm{y}}}_{{\rm{t}}}={{\rm{W}}}_{{\rm{y}}}\times {{\rm{h}}}_{{\rm{t}}}+{{\rm{b}}}_{{\rm{y}}}$$

**Fig. 13:**
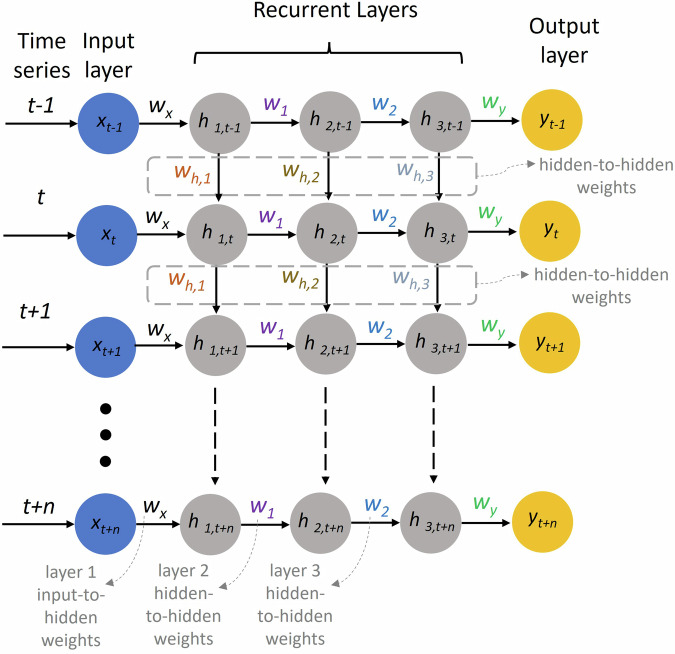
RNN architecture. Schematic of the detailed RNN workflow indicating how each hidden state (highlighted in gray) depends on the previous hidden state, capturing the temporal dependencies in the data.

Figure 13 depicts the architecture and operation of a RNN across multiple time steps. The inputs at different time steps (*x*_*t-1*_, *x*_*t*_, *x*_*t+1*_, …, *x*_*t+n*_) are represented by the blue circles on the left. Each input is processed through several hidden layers (*h*_*1*_, *h*_*2*_, *h*_*3*_) at each time step *t*. The hidden layers are shown by the gray circles. The states at time *t* (*h*_*t*_) depend on the current input and the hidden state from the previous time step, showing how information is passed through time. The weights from input to hidden layers are represented as *w*_*x*_, *w*_*1*_, and *w*_*2*_. The hidden-to-hidden weights are shown as *w*_*h,1*_, *w*_*h,2*_, and *w*_*h,3*_, indicating how the hidden state from one time step influences the next. The output weights are shown as *w*_*y*_. The network produces outputs at each time step (*y*_*t-1*_, *y*_*t*_, *y*_*t+1*_, …, *y*_*t+n*_), represented by the yellow circles on the right. RNNs are a class of DL models, made of artificial neurons with one or more feedback loops. They can be trained on labeled sequential data, where the network learns to predict an output sequence given an input sequence^50^.

One of the limitations with the RNN is the vanishing gradient issue, which affects the effectiveness of this method^52^. To overcome this problem long short-term memory (LSTM)^53^ and gated recurrent units (GRUs)^54^ which are popular RNN architectures and also used to compare their classification accuracies. In this study, TensorFlow libraries^55^ are used to train RNN models.

### Long Short-Term Memory (LSTM)

To address the vanishing gradient issue in Simple RNN models, LSTM networks update hidden states with extra learning parameters, including the forget gate *f*_*t*_, input gate *i*_*t*_, output gate *o*_*t*_, and cell state *c*_*t*_. These values can be calculated using the following equations^56^:

3 $${f}_{t}=\sigma \left({W}_{{if}}{x}_{t}+{b}_{{if}}+{W}_{{hf}}{h}_{t-1}+{b}_{{hf}}\right)$$

4 $${i}_{t}=\sigma \left({W}_{{ii}}{x}_{t}+{b}_{{ii}}+{W}_{{hi}}{h}_{t-1}+{b}_{{hi}}\right)$$

5 $${g}_{t}=\mathrm{tanh}\left({W}_{{ig}}{x}_{t}+{b}_{{ig}}+{W}_{{hg}}{h}_{t-1}+{b}_{{hg}}\right)$$

6 $${o}_{t}=\sigma \left({W}_{{io}}{x}_{t}+{b}_{{io}}+{W}_{{ho}}{h}_{t-1}+{b}_{{ho}}\right)$$

7 $${c}_{t}={f}_{t}\,\odot \,{c}_{t-1}+{i}_{t}\,\odot \,{g}_{t}$$

8 $${h}_{t}={o}_{t}\,\odot \,\tanh ({c}_{t})$$

Where *h*_*t*_ represents the hidden state at time *t*, *c*_*t*_ denotes the cell state at time *t*, and *x*_*t*_ is the input at time *t*. Similarly, *h*_*t-1*_ refers to the hidden state at the previous time step *t-1* or the initial hidden state at time 0. The symbols *i*_*t*_, *f*_*t*_, *g*_*t*_, and *o*_*t*_ correspond to the input, forget, cell, and output gates, respectively. Here, *σ* is the sigmoid activation function, and ʘ represents the element-wise Hadamard product^56^.

### Gated Recurrent Unit (GRU)

The GRU model also addresses the vanishing gradient problem, offering performance similar to LSTM by utilizing a gated structure. However, GRU requires fewer variables and applies a multi-layer gated recurrent unit RNN to process an input sequence. For each item in the input sequence, each layer performs the following function^57^:

9 $${r}_{t}=\sigma \left({W}_{{ir}}{x}_{t}+{b}_{{ir}}+{W}_{{hr}}{h}_{t-1}+{b}_{{hr}}\right)$$

10 $${z}_{t}=\sigma \left({W}_{{iz}}{x}_{t}+{b}_{{iz}}+{W}_{{hz}}{h}_{t-1}+{b}_{{hz}}\right)$$

11 $${n}_{t}=tanh\,({W}_{in}{x}_{t}+{b}_{in}+{r}_{t}\,\odot \,({W}_{hn}{h}_{t-1}+{b}_{hn}))$$

12 $${h}_{t}=(1-{z}_{t})\,\odot \,{n}_{t}+{z}_{t}\,\odot \,{h}_{(t-1)}$$

Where the terms *r*_*t*_, *z*_*t*_, and *n*_*t*_ correspond to the reset, update, and new gates, respectively.

### Long Short-Term Memory (LSTM) autoencoder

Autoencoders are unsupervised representation learning techniques that define non-linear encoder and decoder functions to compress and reconstruct data^58^. LSTM networks can be used in autoencoders to capture temporal dependencies or early anomaly detection in sequential data. LSTM autoencoder extracts the features from the database by reducing the dimensions in the encoding layers. This model is trained by reducing the difference between the original input and the reconstructed data in the decoding layers.

### Random forest (RF)

RF method is an ensemble learning approach that combines predictions from several decision trees by aggregating their outputs^59^. This technique generally shows strong performance in generalizing to unseen data. In this paper, this method is used to compare its performance as a classical ML model with RNN models, as it has a wide application in classification tasks. It is implemented using scikit-learn^47^, and some key hyperparameters are tuned, including the number of trees in the forest (n_estimators), the maximum tree depth (max_depth), the minimum number of samples required to split an internal node (min_samples_split), the minimum number of samples required to be at a leaf node (min_samples_leaf), and the bootstrapping is used (bootstrap). The optimized values of these hyperparameters are reported in the results and discussion section.

### K-means clustering

K-means clustering is an unsupervised technique that classifies the data based on their similarities^60^. This technique associates each input with a label from 1 to k, and it introduces centroids (µ_1_, …, µ_k_), then adjusts both the centroids and the cluster assignments until each input is close to its assigned centroid^61^. In this study, the output features extracted by the LSTM autoencoder are further reduced using PCA and then clustered using the K-means algorithm. The number of clusters is set to *k* = *4*, which reflects the predefined classification structure. The K-means model was implemented using scikit-learn’s K-means class with a fixed random state (random_state=0) to ensure reproducibility. After fitting the model on the PCA-transformed training data, cluster assignments are predicted and the corresponding centroids are extracted.

### Hyperparameter tuning

Hyperparameter tuning refers to the process of optimizing the performance of a ML model by selecting the best values for hyperparameters. Unlike parameters that the model learns during training, hyperparameters are set prior to training and determine the overall behavior of the model^62^. In the present study, two techniques are used for hyperparameter tuning which are Bayesian hyperparameter optimization and grid search.

The grid search technique searches through a predefined grid of hyperparameter combinations^63^. Each combination is tested by training the model and evaluating its performance, using cross validation. Grid search is deployed for tuning the depth and number of estimators in the RF model.

Bayesian optimization builds a probabilistic model of the objective function, such as validation accuracy, and uses that model to decide where to evaluate the next set of hyperparameters^64^. Such Bayesian based approach aims to find the optimal hyperparameters with fewer evaluations compared to grid search, making it faster and more computationally feasible^65^. This method is useful when tuning DL models or models with many hyperparameters, such as the number of layers and units in RNN. Bayesian optimization reduces the number of trials by focusing the search on promising regions of the hyperparameter space based on previous evaluations, making it suitable for scenarios where model training is computationally expensive.

### Evaluation of the learning models

To evaluate model performance, k-fold cross-validation with three folds is employed using the KFold method from sklearn.model_selection. This process is done to ensure that overfitting is not occurred to a single training set^66^. To evaluate the classification performance of the models, the confusion matrix, accuracy score, F1-score, precision, and recall are typically calculated for each model^67^. All these metrics are therefore adopted in the present study and are computed using the test data that the models have not seen during training.

The accuracy represents the proportion of correctly predicted labels out of the total number of predictions and is calculated using Eq. 13^68^.

13 $${Accuracy}=\,\frac{{TP}+{TN}}{{TP}+{TN}+{FP}+{FN}}$$

Where TP denotes the true positives, i.e., correctly predicted positive instances, TN represents the true negatives, i.e., correctly predicted negative instances, FP denotes the false positives, i.e., incorrectly predicted positive instances, and FN presents the false negatives, i.e., incorrectly predicted negative instances.

Precision is the proportion of the TP predictions out of all positive predictions made by the model as shown in Eq. 14^68^. As such, a high precision indicates that the model makes only few false positive errors.

14 $${Precision}=\,\frac{{TP}}{{TP}+{FP}}$$

Recall measures the proportion of actual positives that are correctly identified as calculated in Eq. 15^68^, and high recall means the model captures most of the positive instances, but it might also include more false positives.

15 $${Recall}=\,\frac{{TP}}{{TP}+{FN}}$$

The F1-score is the harmonic mean of precision and recall and is calculated using Eq. 16^68^. It balances the two metrics and is particularly useful when dealing with unbalanced classes. A high F1-score indicates that the model has both good precision and recall, making it an effective overall measure of model performance.

16 $$F1-{Score}=2\times \,\frac{{Precision}\times {Recall}}{{Precision}+{Recall}}$$

A confusion matrix is a table used to evaluate the performance of a classification model on a test dataset with known true values. It has two dimensions: one indexed by the actual class and the other by the predicted class provided by the classifier^69^. It provides the counts of TPs, FPs, TNs, and FNs.

## Data Availability

The used raw data and developed codes required to reproduce these findings are available to download from the following Github repository: https://github.com/Soroosh-HKN/ECN-RNN.
